# Supplementary material for: Study protocol for evaluating the effectiveness of depression management on gylcaemic control in non-communicable diseases clinics in Malawi
Source: BMJ Open. 2018 Oct 15;8(10):e021601. doi: 10.1136/bmjopen-2018-021601 (PMC6194461; doi:10.1136/bmjopen-2018-021601)
Supplement: Author's manuscript [file bmjopen-2018-021601.draft_revisions.pdf]

BMJ Open

BMJ Open is committed to open peer review. As part of this commitment we make the peer review history of every article we publish publicly available.

When an article is published we post the peer reviewers' comments and the authors' responses online. We also post the versions of the paper that were used during peer review. These are the versions that the peer review comments apply to.

The versions of the paper that follow are the versions that were submitted during the peer review process. They are not the versions of record or the final published versions. They should not be cited or distributed as the published version of this manuscript.

BMJ Open is an open access journal and the full, final, typeset and author-corrected version of record of the manuscript is available on our site with no access controls, subscription charges or pay-per-view fees (http://bmjopen.bmj.com).

If you have any questions on BMJ Open's open peer review process please email editorial.bmjopen@bmj.com

BMJ Open

The effectiveness of depression management on diabetes outcomes in Malawi - a study protocol

Journal:	*BMJ Open*
Manuscript ID	bmjopen-2018-021601
Article Type:	Protocol
Date Submitted by the Author:	10-Jan-2018

Complete List of Authors:	Udedi, Michael; University of Malawi College of Medicine, Mental Health Department; Ministry of Health, Malawi, Clinical Services<br>Pence, B; University of North Carolina at Chapel Hill,<br>Kauye, F.; University of Malawi College of Medicine, Mental Health Department<br>Muula, Adamson S.; University of Malawi College of Medicine, Department of Public Health, School of Public Health and Family Medicine; University of Malawi College of Medicine, Africa Center of Excellence in Public Health and Herbal Medicine

Keywords:	Diabetes, Depression, Non communicable diseases, depression management, comorbidity

SCHOLARONE™ Manuscripts

For peer review only

The effectiveness of depression management on diabetes outcomes in Malawi - a study protocol

Michael Udedi [1,2,3]* Brian W. Pence[4], Felix Kauye [1,3], Adamson S. Muula [3,5]

[1] University of Malawi
College of Medicine,
Department of Mental Health
P/Bag 360
Chichiri
Blantyre 3, Malawi
mphatsoudedi@yahoo.co.uk
felixkauye@yahoo.com

[2]Ministry of Health,
Lilongwe,
Malawi

[3]Department of Public Health,
School of Public Health and Family Medicine,
University of Malawi,
College of Medicine,
Blantyre, Malawi

[4] University of North Carolina at Chapel Hill
Department of Epidemiology
McGavran-Greenberg 2103C
Campus Box 7435
UNC-Chapel Hill
Chapel Hill, NC 27599-7435
bpence@unc.edu

[5]Africa Center of Excellence in Public Health and Herbal Medicine,
University of Malawi,
College of Medicine,
Blantyre, Malawi
amuula@medcol.mw

*Corresponding author:
Michael Udedi,
University of Malawi,
College of Medicine,
P/Bag 360,
Chichiri, Blantyre 3, Malawi
E-mail: mphatsoudedi@yahoo.co.uk

For peer review only

ABSTRACT

Introduction: Depression is associated with negative patient outcomes for chronic diseases and likely affects consistent physical non-communicable diseases (NCDs) care management in relation to clinic attendance and medication adherence. We found no published studies on the integration of depression management in physical NCD clinics in Malawi and assessing its effects on patient and service outcomes. Therefore, the aim of this study is to evaluate the effectiveness of integrating depression screening and management in physical NCD routine care on patient and service outcomes in Malawi. We will also determine the sensitivity and specificity of the PHQ-9 in the detection of depression in NCDs clinic.

Methods and Analysis: The study will have two phases. Phase one will involve the validation of the PHQ-9 screening tool for depression, using a cross sectional study design involving 323 participants, in two specialised physical NCD clinics in one of the 28 districts of Malawi. Using a quasi-experimental study design in four districts of Malawi not involved in the Phase one study, Phase two study will evaluate the effectiveness of integrating depression screening (using PHQ-9) and management (based on a specially designed toolkit). Outcomes will be measured at three and six months among patients with comorbid diabetes (poorly controlled) and depression attending physical NCD clinics in Malawi.

Ethics and Dissemination: Ethical approval was obtained from the University of Malawi, College of Medicine Research and Ethics Committee (COMREC). The findings will be disseminated through presentations at journal clubs, senior management of the Ministry of Health, national and international conferences as well as submission to peer reviewed publications. Policy briefs will also be created.

For peer review only

Strengths and limitations of this study

- The cross sectional study design in phase one will allow us to estimate the prevalence of depression among people with diabetes**.**
- The quasi experimental design will enable us to investigate the impact of depression in determining the outcome of diabetes.
- The quasi-experimental design which will be used in second phase has a limitation that baseline differences between the clinics may lead to spurious results.

For peer review only

INTRODUCTION

Physical non communicable diseases and depression individually are common and significant causes of morbidity, disability and mortality in Sub Saharan Africa [1]. Non communicable diseases (NCDs) account for a growing burden on the population and health systems in low-and middle-income countries (LMIC), [2]. Similarly depression is projected to become a leading cause of disability by 2030 [3]. The World Health Survey of 2007 suggests that the prevalence of comorbidity of physical NCDs and depression is increasing; thus between 9.3% and 23.0% of people with NCDs had comorbid depression worldwide [4]. The global prevalence of major depression in people with diabetes is 27% [5]. The relationship between depression and physical NCDs is bidirectional. Modifiable risk factors for physical NCDs are exacerbated by poor mental health and on the other hand physical NCDs are a risk factor for mental illness[6]. Comorbidity is associated with a range of outcomes, including poor adherence or non-adherence to medication, functional impairment, increased medical costs, poor self-care regimens, increased medical symptom burden, increased morbidity and increased mortality [7 8 9].

Physical NCDs are common and in Malawi, it is estimated that NCDs account for at least 12% of total Disability Adjusted Life Years (DALYs) [10]. In addition, NCDs are the second leading cause of deaths in adults after AIDS [10]. Furthermore, a 2009 nationwide STEPwise approach to Surveillance (STEPS) survey showed that about 6% of the general population had diabetes mellitus [11].

Depression commonly overlaps with physical NCDs and depression complicates NCD care. We are not aware of any studies done in Malawi that assessed the burden of depression in physical NCDs care and its effect on diabetes outcomes. A recent study done in one of the 28 districts of Malawi, reported that control of diabetes is poor and there was high non adherence of NCDs clients to appointments [12]. Currently there are no published studies done in Malawi to explore factors associated with poor control of diabetes and the high rate of non-adherence to attending appointments in physical NCDs clinics.

In Malawi, there are few studies looking at depression screening and treatment in general care. The prevalence of depression among outpatient attendees in primary care clinics in Malawi in 2010 and 2013 ranged between 19 to 30% [13 14]. Despite the high prevalence of depression in general health care services in Malawi, the extent of comorbid depression in the context of NCDs is unclear. Using the results from general primary care clinics studies done in Malawi on detection rates for depression, it is likely that detection rates of depression in physical NCDs clinics in Malawi are poor. Undiagnosed and untreated depression more likely contributes to

For peer review only

poor control of diabetes as shown in studies done in other countries [15-17]. Routine screening will assist in early detection of patients with co-morbid depression in physical NCD clinics and facilitate treatment. To our knowledge, there are no studies done in Malawi focusing on identification and interventions for depression in physical NCDs clinics.
The integration of mental healthcare into other health services is one of the strategies in the National Mental Health Policy in Malawi[18]. The results of the proposed study will help to inform on decisions whether to integrate and the strategies for integrating depression management in physical NCD clinics in Malawi.

AIMS AND OBJECTIVES

Our overall objective is to evaluate the effectiveness of integrating screening and depression management into physical NCD routine care on patient and service outcomes in Malawi. In this way, we will contribute to evidence on comorbidity of depression and diabetes including the effects of identifying and treating depression in patients with comorbid diabetes and depression in physical NCD care in Malawi.

Our study has four specific objectives:

1. To determine the sensitivity and specificity of the PHQ-9 in the detection of depression in NCDs clinic,
2. To estimate the prevalence of comorbidity between depression and diabetes mellitus in Malawi,
3. To determine detection rate of depression in physical NCDs clinics in Malawi,
4. To compare patient outcomes in physical NCDs clinics with and without integration of depression screening and management.

RESEARCH QUESTIONS

The main research questions in this study are:

1. Will the validated Chichewa Patient Health Questionnaire (PHQ-9) demonstrate satisfactory sensitivity and specificity in physical NCD clinic settings in Malawi?
2. Will integrating depression screening using a validated PHQ-9 and depression management (based on a specially designed toolkit) be more effective than usual care in identifying patients with comorbid depression and diabetes, facilitating treatment of depression in patients with comorbid depression and diabetes, and improving clinical outcomes among patients with comorbid depression and diabetes?

For peer review only

METHODS AND ANALYSIS

The study will be conducted from December 2017 to December 2018. The study will be in two phases. Phase One will be for validation of the PHQ 9 among diabetes patients. Phase two will be evaluating the effectiveness of integrating screening and depression management in patients with comorbid depression and diabetes attending physical NCD clinics. To answer the research questions, we will use a cross-sectional study design in Phase One and a quasi-experimental study design in Phase Two.

Phase One: Validation of PHQ 9 in physical NCDs Clinic Setting in Malawi

Study design, area and physical NCD clinics

For Phase One, we will use a cross sectional study design. The study will be conducted in Lilongwe district which is one of the 28 districts in Malawi. Malawi is divided administratively into three regions namely Northern, Central and Southern. Lilongwe is located in the central region of Malawi. It has a population of 2,688, 379 people[19] with 8 physical NCD clinics.

The study will be conducted in two physical NCDs clinics (Area 25 Health Centre under Lilongwe District Health Office and Kamuzu Central Hospital). The Area 25 Health Centre serves a catchment population of 80, 000 while Kamuzu Central Hospital (a tertiary health facility) catchment population is the entire central region which is 7, 401,068 [19]. The physical NCD clinic at Area 25 Health Centre is staffed by clinical officers and nurses who have undergone appropriate training in the diagnosis and management of key NCDs while the clinic of Kamuzu Central Hospital is also staffed by medical officers (general medical doctors) and specialists on top of clinical officers and nurses. The two sites are implementing the World Health Organization Package of Essential Non communicable diseases (WHO PEN) interventions. Figure 1 is a map of Malawi showing central region districts

(Insert figure 1 here)

SAMPLE SIZE DETERMINATION

The sample size was calculated based on sensitivity of PHQ 9 using Buderer's formula [20]. We used the following parameters; anticipated sensitivity (*SN*) was 80%, standard normal deviation corresponding to the specified size of the critical region ($z^2_{1-\alpha/2}$) is 3.84, alpha (α) which is size of the critical region is 0.05, L is the absolute precision desired on either side of sensitivity and was set at 0.1 and prevalence for depression was 20%. The prevalence of depression of 20% is based on a recent study on patients attending health care setting in Malawi [13]. We also

For peer review only - http://bmjopen.bmj.com/site/about/guidelines.xhtml

considered potential participants' refusal and loss of data; consequently we put it at 5%. Taking these assumptions into consideration, the required sample size was calculated as 323.

RECRUITMENT AND DATA COLLECTION

Consecutive patients attending the NCD Clinic will be identified by research assistants. The research assistants will provide information about the study to the patients and seek informed written consent. Participants will be eligible for the study if they are at least 18 years of age and attending the NCDs clinic for diabetes care and available for an interview. Participants will be excluded if they require acute medical care or are unable to speak.

The research assistants will enrol patients who consent and carry out interviews at the health facility. When patients do not consent to participate in the study, the research assistant will request permission to record sex, age, level of education and occupation of the patient.
One research assistant will administer the PHQ 9 orally to ensure high and standard responses, due to low literacy of the study participants. The research assistant will then calculate the screening score and let the participant be attended to by the NCDs clinician. After the consultation the patient will have the Diagnostic and Statistical Manual of Mental Disorders (DSM–IV), Axis I disorders Clinical Version (SCID) administered by a second research assistant to elicit the psychiatric symptoms itemized in the Diagnostic and Statistical Manual of Mental Disorders (DSM) criteria for depression. The second research assistant will be masked from the PHQ-9 score. The training, supervision, and quality assurance of the research assistant doing the SCID will be done as well. A diagrammatic representation of the process is shown below in Figure 2.

(Insert figure 2 here)

MEASUREMENTS

The aim of Phase One is validating the Patient Health Questionnaire (PHQ-9) in physical NCDs clinics in order for it to be used for screening of depression in NCDs in Malawi. The PHQ-9 (based on DSM criteria) has been chosen for this study because it is brief, requires a short time to administer which is compatible with the Malawian health setting workload. Furthermore, the PHQ-9 can be used to establish depressive disorder diagnoses as well as depressive symptoms severity [21].

For screening data using PHQ-9, presence of depressive symptoms will be defined as having a total score of 5 and above on all the nine questions (PHQ-9 total score ≥ 5). Symptoms will be categorised using established cut-off points 5, 10 and 20 (mild, moderate, and severe depressive symptoms, respectively) [22 23]. The sensitivity and specificity at the standard cut-off

For peer review only - http://bmjopen.bmj.com/site/about/guidelines.xhtml

point will be assessed, and performance at alternative cut-off point will also be examined to determine whether the standard cut-off point is optimal in this population

The SCID for depression will be used as the Gold standard to validate PHQ 9. The SCID for depression has been translated into Chichewa and has been used in Malawi after undergoing a process of validation which included translation, back translation and testing [24] .

Data management

Data will be checked for consistency and completeness by the principal investigator and double entered to SPSS V.20 by experienced data entry clerks. Then, it will be cleaned and analysed using the same SPSS V.20. Hard copies of the data will be stored in a locked cabinet and consent forms will be separated from the data.

Data analysis

Data will be analysed using SPSS V.20. Descriptive statistics will be computed to describe the sociodemographic characteristics of participants and to summarise the frequencies of depression under each category in both criteria (PHQ-9 and SCID). We will assess whether refusal is associated with sociodemographic characteristics by testing the statistical significance of socio-demographic characteristics between refusals and consenters. The prevalence of diagnosed depression among patients with diabetes will be determined by computing the proportion of those with depression according to the SCID who will be correctly screened out by the PHQ.

The sensitivity, specificity and predictive values (PPV and NPV) of a PHQ-9 total score >=5, with 95% confidence intervals, will be calculated relative to the reference standard of a diagnosis from the depression module of the SCID. A Receiver Operating Characteristic curve (ROC curve) will be made using the SPSS version 20 in order to get criterion validity of the PHQ-9. Internal consistency will be determined by Cronbach's alpha. In exploratory analyses we will examine the test characteristics of alternative thresholds.

Phase Two: Evaluation of the effectiveness of integrating screening using validated PHQ 9 and Depression management in physical NCD Clinics

Study design and setting

In Phase Two of the study, we will use a quasi experimental design employing a non-equivalent control group (pre-post) design. Prospective controlled interventional trials provide a higher level of evidence for a true causality [25]. The best choice of the controlled interventional

For peer review only - http://bmjopen.bmj.com/site/about/guidelines.xhtml

studies is the true experimental design of which randomized controlled trial (RCT) is the gold standard. However nonrandomized control group trial is a reasonable choice when an RCT would be ideal but not due to costs and ethics limitations and also when trying to establish the effectiveness of large-scale dissemination and implementation [25 26]. Furthermore literature indicates the intervention often cannot be randomized for the following reasons: ethical considerations, an inability to randomize patients, an inability to randomize locations or settings, and a need to intervene quickly[26]. We have therefore chosen to use a quasi experimental design and the intervention program will be done in two district NCD clinics while the other two districts will provide usual care. Four convenient physical NCD clinics which are implementing the World Health Organization Package of Essential Non-communicable diseases (WHO PEN) interventions will be allocated to intervention arm (2 physical NCD clinics) and control arm (2 physical NCD clinics) and in each clinic, consecutive patients who meet the study criteria will be recruited.

The Phase Two of the study will be conducted for a period of 9 to 12 months in four health care facilities in Mchinji district, Kasungu district, Salima district and Dedza district of the Central region of Malawi (Figure 1). The four districts are also implementing the World Health Organization Package of Essential Non communicable diseases (WHO PEN) interventions. Currently each district has one physical NCD Clinic based at the District Hospital. The physical NCD clinic in the four districts are staffed by clinical officers and nurses who have undergone appropriate training in the diagnosis and management of key physical NCDs. They follow similar standard guidelines in the management of the physical NCDs.

Intervention

1. Clinicians at the physical NCD clinics will be trained in routine screening for depression with a validated PHQ 9,
2. Clinicians at the physical NCD clinics will be trained on comorbidity of diabetes and depression and coming up with individual care plans and prescribing and monitoring antidepressants
3. Clinicians at the physical NCD clinics will be trained on problem solving skills using the effective helping techniques for depression and how to make follow up on individual care plans.

Usual Care

This would involve the NCD clinicians treating patients with comorbid depression and diabetes using normal practices of treating depression involving antidepressants.

For peer review only - http://bmjopen.bmj.com/site/about/guidelines.xhtml

SAMPLE SIZE DETERMINATION

The sample size was based on detecting a difference in glycated haemoglobin at 6 months between study arms. The sample size was based on an Analysis of Covariance (ANCOVA) approach, with the outcome measurement at follow-up, and the baseline glycated haemoglobin considered as a covariate in the analysis. The correlation between baseline and follow-up haemoglobin values is estimated to be 0.5. A difference in glycated haemoglobin between arms of 0.7% is considered to be a clinically important difference (based on a previous study which found a difference of 0.56% [27]), and data from previous research suggested that the glycated haemoglobin measurements had a standard deviation of 1.8% [27]. It is calculated that using a 5% significance level and 80% power, 78 subjects per group are required. It is estimated that there will be a 10% drop out/loss to follow-up. To allow for this, we planned to recruit 87 per group, giving a total sample size for the study of 174 participants.

RECRUITMENT AND DATA COLLECTION

We will use a mixed methods (quantitative and qualitative) approach in Phase two. The reason for collecting both quantitative and qualitative data is to complement and also to address shortfall s of either approach. The use of both forms of data allows researchers to gain deeper understanding of the phenomenon under study [28]. In addition, evidence suggests that triangulation of methods increases both the validity and reliability of data [29].

The research assistant with the help of health professionals running the NCDs clinic at each site will identify the study participants who meet the inclusion criteria consecutively. The research assistants will provide information about the study to the patient and seek written informed consent. Participants will be eligible for the study if they are at least 18 years of age and attending the NCDs clinic for diabetes care and available for an interview. Participants will be excluded if they require acute medical care or are unable to speak. The research assistants will enrol patients who consent and carry out interviews at the health facility. When patients do not consent to participate in the study, the research assistant will request permission to record sex, age, level of education and occupation of the patient. Adult patients with poorly controlled diabetes (a glycated hemoglobin level of 8.5% or higher as suggested from previous research [27 30]) and comorbid depression will be recruited for the study and followed up to 6 months after recruitment (Figure 3). Data collection will occur at three time-points: baseline, at 3 months and at 6 months.

Qualitative interviews will be conducted among health care workers in order to assess feasibility and acceptability of integrating depression management into NCDs care. In-depth

For peer review only - http://bmjopen.bmj.com/site/about/guidelines.xhtml

interviews will be employed to understand health professionals' experience in both using the validated PHQ-9 for screening and the depression management guidelines.

(Insert figure 3 here)

MEASUREMENTS

The primary exposure variable

Depression will be measured using the PHQ-9. The PHQ-9 will have been validated in Phase One of this study.

The exposure of interest is whether the patient is receiving care at an intervention or usual care facility.

Outcome variables

Primary outcome variable

The patient diabetes outcome for this study is blood glucose control. Data on the patients' status on the primary outcome will be extracted from the NCD patient mastercard. For the purpose of the study in checking how well diabetes is being controlled, glycated haemoglobin (HbA1c) test will be performed on the recruited study participants at baseline, 3 and 6 months.

Secondary outcome variable

Quality of life (QoL) will be measured using the interviewer administered version of the EuroQol 5 dimensions questionnaire (EQ-5D). This instrument has been validated in South Africa and Zimbabwe [31 32]. The tool has been translated into several languages including Afrikaners, Xhosa and Shona [32]. The EQ-5D will be adapted in Malawi and will be used at baseline and on follow up of patients at three and six months.

Data management

Data will be checked for consistency and completeness by the principal investigator and double entered to SPSS V.20 by experienced data entry clerks. Hard copies of the data will be stored in a locked cabinet and consent forms will be separated from the data.

Data analysis

The primary outcome is glycated haemoglobin at 6 months. This will be compared between groups using Analysis of Covariance (ANCOVA). The haemoglobin value at baseline will be considered as a covariate in the analysis.

For peer review only

The glycated haemoglobin values at 3 months will be considered as a separate outcome, and analysed using equivalent methods to the primary outcome.

Quantitative data will be analyzed statistically using a statistical package for social sciences version 20 (SPSS-20). Descriptive analysis will be done. The recorded qualitative data on the feasibility and acceptability of integrating depression management will be transcribed and coded using Atlas ti. Relevant queries will be created in the database to thematically analyse the data.

Ethical considerations

The protocol was approved by the University of Malawi, College of Medicine Research and Ethics Committee (COMREC) on 31st August 2017 (Reference-P.07/17/2218). Written informed consent will be obtained from every participant and fingerprint impressions will be taken from consenting illiterate participants. Patients identified as having depression or endorsing the suicide item on PHQ-9, will be referred to appropriate professionals for further assessment and management. A permission to conduct the study has been sought from the District Health Officers of Lilongwe, Kasungu, Dedza, Mchinji, Salima and Hospital Director of Kamuzu Central Hospital. Study participants will not be given any financial reimbursement, however they will thanked verbally for their participation. Privacy will be maintained as the interviews will be conducted in a room which will provide both visual and audio privacy. Referrals will be made to appropriate professionals for further assessment and management in cases where participants will be identified to have depression as well as those that will screen positive for suicidal ideations through the interviews.

Dissemination plan

The findings will be disseminated through publications in peer-reviewed journals and conference presentations. Summary reports will be submitted to the participating institutions and to the University of Malawi, College of Medicine. The results will also be shared with policy makers and other local stakeholders in form of policy briefs as part of evidence on integration of depression management in NCDs.

Limitations of the study

In this study, people may have undiagnosed comorbid illnesses and our method of screening may not be strong in capturing other comorbid illnesses. Quality of life will be measured using five domains and we will therefore not obtain detailed information on the various dimensions that assess health-related quality of life (HRQoL). The quasi-experimental design which will be

For peer review only

used in second phase has also a limitation that baseline differences between the clinics may lead to spurious results.

Strengths of the study

The study will provide much needed evidence about the impact of comorbid depression on the outcome of diabetes. The cross sectional study design will allow us to estimate the prevalence of depression among people with diabetes. The study will enable us to investigate the impact of depression in determining the outcome of diabetes. To our knowledge, this study will be the first in Malawi, to examine identification and interventions for depression in physical NCD clinics.

Expected benefits of the findings

Our findings will contribute to sparse evidence on mental health, diabetes and other NCDs in low- income and middle-income countries. We anticipate that our study findings will raise awareness of comorbidity among clinicians, service providers, policy makers and that it will contribute to clinical practice and policy change regarding the delivery of mental healthcare in NCDs care in Malawi.

Authors' contributions

MU, BP, FK and AM were involved in the conceptualization of the study. MU was responsible for drafting the protocol manuscript. BP, FK and AM edited the protocol. All authors read and approved the final manuscript.

Funding statement

This study protocol presents independent work supported through the DELTAS Africa Initiative [DEL-15-01]. The DELTAS Africa Initiative is an independent funding scheme of the African Academy of Sciences (AAS)'s Alliance for Accelerating Excellence in Science in Africa (AESA) and supported by the New Partnership for Africa's Development Planning and Coordinating Agency (NEPAD Agency) with funding from the Wellcome Trust [DEL-15-01] and the UK government. The views expressed in this publication are those of the author(s) and not necessarily those of AAS, NEPAD Agency, Wellcome Trust or the UK government.

Competing interests statement

The authors declare that they have no competing interests.

For peer review only

Reference

1. Nikolic IA, Stanciole AE, Zaydman M. Chronic emergency: why NCDs matter. Health, Nutrition, and Population Discussion Paper 2011.
2. Buttorff C, Hock RS, Weiss HA, et al. Economic evaluation of a task-shifting intervention for common mental disorders in India. Bulletin of the World Health Organization 2012;**90**(11):813-21.
3. Mathers C, Fat DM, Boerma JT. The global burden of disease: 2004 update. Geneva, Switzerland: World Health Organization. 2008.
4. Moussavi S, Chatterji S, Verdes E, et al. Depression, chronic diseases, and decrements in health: results from the World Health Surveys. The Lancet 2007;**370**(9590):851-58.
5. World Health Organization. Investing in mental health. Geneva: World Health Organization, 2003.
6. Ngo VK, Rubinstein A, Ganju V, et al. Grand challenges: integrating mental health care into the non-communicable disease agenda. PLoS Med 2013;**10**(5):e1001443.
7. Mensah GA, Collins PY. Understanding mental health for the prevention and control of cardiovascular diseases. Global heart 2015;**10**(3):221-4.
8. Rieckmann N, Gerin W, Kronish IM, et al. Course of depressive symptoms and medication adherence after acute coronary syndromes: an electronic medication monitoring study. J Am Coll Cardiol 2006;**48**(11):2218-22.
9. Gehi A, Haas D, Pipkin S, et al. Depression and medication adherence in outpatients with coronary heart disease: findings from the Heart and Soul Study. Arch Intern Med 2005;**165**(21):2508-13.
10. MoH, WHO. Malawi National STEPS Survey for Chronic Non-Communicable Diseases and their Risk Factors, 2010.
11. Msyamboza KP, Ngwira B, Dzowela T, et al. The burden of selected chronic non-communicable diseases and their risk factors in Malawi: nationwide STEPS survey. PLoS One 2011;**6**(5):e20316.
12. Manjomo RC, Mwagomba B, Ade S, et al. Managing and monitoring chronic non-communicable diseases in a primary health care clinic, Lilongwe, Malawi. Public Health Action 2016;**6**(2):60-65.
13. Kauye F, Jenkins R, Rahman A. Training primary health care workers in mental health and its impact on diagnoses of common mental disorders in primary care of a developing country, Malawi: a cluster-randomized controlled trial. Psychological medicine 2014;**44**(03):657-66.
14. Udedi M. The prevalence of Depression among patients and its detection by primary health care workers at Matawale Health Centre (Zomba). Malawi Medical Journal 2014;**26**(2):34-37.
15. Ciechanowski P, Russo J, Katon W, et al. Where is the patient? The association of psychosocial factors and missed primary care appointments in patients with diabetes. General hospital psychiatry 2006;**28**(1):9-17.
16. Andreoulakis E, Hyphantis T, Kandylis D, et al. Depression in diabetes mellitus: a comprehensive review. Hippokratia 2012;**16**(3):205-14.
17. Lin EHB, Rutter CM, Katon W, et al. Depression and Advanced Complications of Diabetes. Diabetes Care 2010;**33**(2):264.
18. Ministry of Health and Population. National Mental Health Policy. Lilongwe, Malawi: Ministry of Health and Population, 2001.
19. National Statistical Office (NSO). Population Projections Malawi. Zomba, Malawi: National Statistical Office (NSO), 2009.
20. Buderer NMF. Statistical methodology: I. Incorporating the prevalence of disease into the sample size calculation for sensitivity and specificity. Academic Emergency Medicine 1996;**3**(9):895-900.
21. Kroenke K, Spitzer RL, Williams JBW. The PHQ-9: Validity of a Brief Depression Severity Measure. Journal of General Internal Medicine 2001;**16**(9):606-13.
22. Lowe B, Kroenke K, Herzog W, et al. Measuring depression outcome with a brief self-report instrument: sensitivity to change of the Patient Health Questionnaire (PHQ-9). Journal of affective disorders 2004;**81**(1):61-66.
23. Lowe B, Unutzer J, Callahan CM, et al. Monitoring depression treatment outcomes with the patient health questionnaire-9. Medical care 2004;**42**(12):1194-201.
24. Stewart RC, Kauye F, Umar E, et al. Validation of a Chichewa version of the self-reporting questionnaire (SRQ) as a brief screening measure for maternal depressive disorder in Malawi, Africa. Journal of Affective Disorders 2009;**112**(1-3):126-34.

For peer review only

25. Axelrod DA, Hayward R. Nonrandomized Interventional Study Designs (Quasi-Experimental Designs). In: Penson DF, Wei JT, eds. Clinical Research Methods for Surgeons. Totowa, NJ: Humana Press, 2007:63-76.
26. Eliopoulos GM, Harris AD, Bradham DD, et al. The use and interpretation of quasi-experimental studies in infectious diseases. Clinical Infectious Diseases 2004;**38**(11):1586-91.
27. Katon WJ, Lin EH, Von Korff M, et al. Collaborative care for patients with depression and chronic illnesses. New England Journal of Medicine 2010;**363**(27):2611-20.
28. Creswell JW. *Research design: Qualitative, quantitative, and mixed methods approaches*: Sage publications, 2013.
29. Ajayi I, Olumide E, Oyediran O. Patient satisfaction with the services provided at a general outpatients' clinic, Ibadan, Oyo State, Nigeria. African journal of medicine and medical sciences 2005;**34**(2):133-40.
30. Wilkinson JE, Culpepper L. Peer Reviewed: Associations Between Colorectal Cancer Screening and Glycemic Control in People With Diabetes, Boston, Massachusetts, 2005-2010. Preventing chronic disease 2011;**8**(4).
31. Jelsma J, Mhundwa K, De Weerdt W, et al. The reliability of the Shona version of the EQ-5D. The Central African journal of medicine 2001;**47**(1):8-13.
32. Jelsma J, Ferguson G. The determinants of self-reported health-related quality of life in a culturally and socially diverse South African community. Bulletin of the World Health organization 2004;**82**(3):206-12.

For peer review only

Figures

Figure 1: Map of Malawi showing central region districts and study sites

Figure 2: Phase One study procedure

Figure 3: Phase Two study procedure

For peer review only

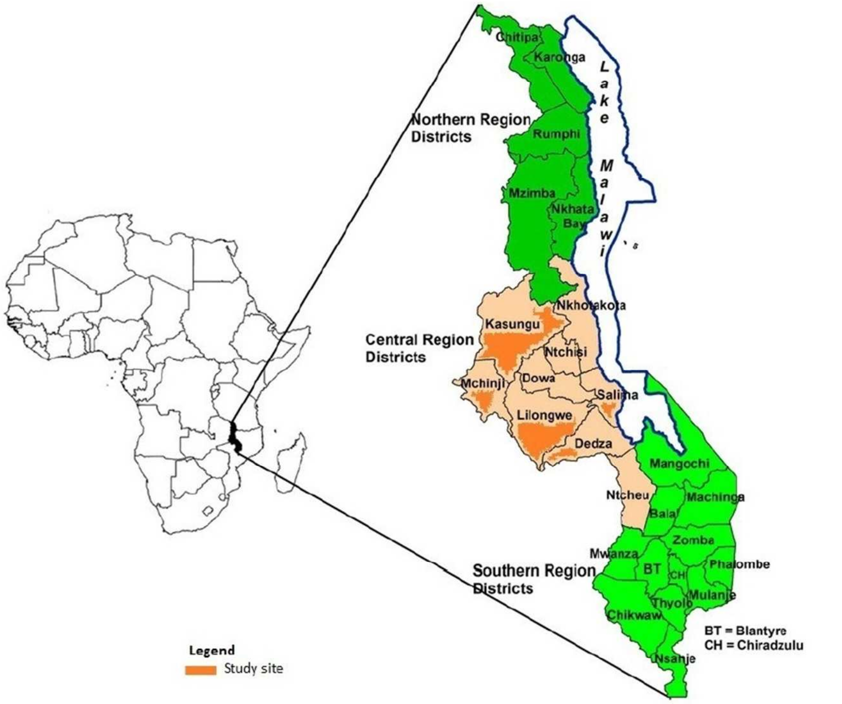

Figure 1: Map of Malawi showing central region districts and study sites

85x65mm (300 x 300 DPI)

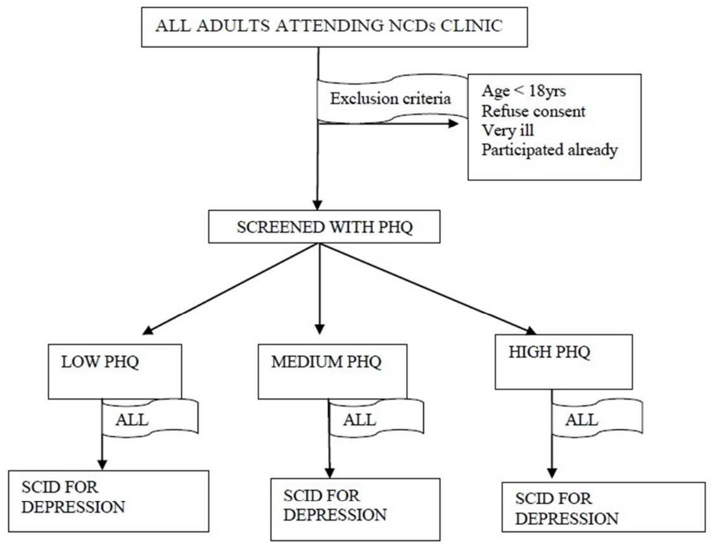

Figure 2: Phase One study procedure

87x56mm (300 x 300 DPI)

view only

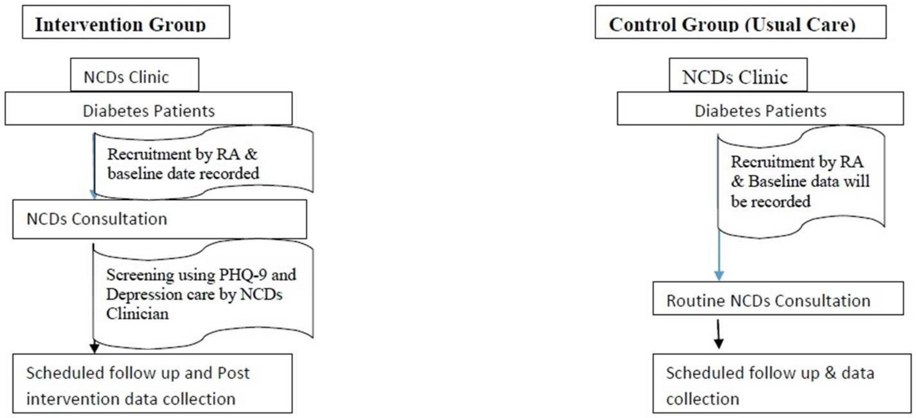

**Three months follow up**		**Three months follow up**
PHQ 9 score		PHQ 9 score
Diabetes control check		Diabetes control check
EQ-5D		EQ-5D
**Six month follow up**		**Six month follow up**
PHQ 9		PHQ 9
Diabetes control check		Diabetes control check
EQ-5D		EQ-5D
**Baseline assessments will be done for all three measures & recorded**		**Baseline assessments will be done for three measures only & recorded**

Figure 3: Phase Two study procedure

88x87mm (300 x 300 DPI)

BMJ Open

Study protocol for evaluating the effectiveness of depression management on gylcaemic control in non communicable diseases clinics in Malawi

Journal:	*BMJ Open*
Manuscript ID	bmjopen-2018-021601.R1
Article Type:	Protocol
Date Submitted by the Author:	18-May-2018

Complete List of Authors:	Udedi, Michael; University of Malawi College of Medicine, Mental Health Department; Ministry of Health, Malawi, Clinical Services<br>Pence, B; University of North Carolina at Chapel Hill,<br>Kauye, F.; University of Malawi College of Medicine, Mental Health Department<br>Muula, Adamson S.; University of Malawi College of Medicine, Department of Public Health, School of Public Health and Family Medicine; University of Malawi College of Medicine, Africa Center of Excellence in Public Health and Herbal Medicine

**Primary Subject Heading**:	Mental health
Secondary Subject Heading:	Diabetes and endocrinology
Keywords:	Diabetes, Depression, Non communicable diseases, depression management, comorbidity

SCHOLARONE™
Manuscripts

Study protocol for evaluating the effectiveness of depression management on gylcaemic control in non communicable diseases clinics in Malawi

Michael Udedi [1,2,3]* Brian W. Pence[4], Felix Kauye [1,3], Adamson S. Muula [3,5]

[1] University of Malawi
College of Medicine,
Department of Mental Health
P/Bag 360
Chichiri
Blantyre 3, Malawi
mphatsoudedi@yahoo.co.uk
felixkauye@yahoo.com

[2]Ministry of Health,
Lilongwe,
Malawi

[3]Department of Public Health,
School of Public Health and Family Medicine,
University of Malawi,
College of Medicine,
Blantyre, Malawi

[4] University of North Carolina at Chapel Hill
Department of Epidemiology
McGavran-Greenberg 2103C
Campus Box 7435
UNC-Chapel Hill
Chapel Hill, NC 27599-7435
bpence@unc.edu

[5]Africa Center of Excellence in Public Health and Herbal Medicine,
University of Malawi,
College of Medicine,
Blantyre, Malawi
amuula@medcol.mw

*Corresponding author:
Michael Udedi,
University of Malawi,
College of Medicine,
P/Bag 360,
Chichiri, Blantyre 3, Malawi
E-mail: mphatsoudedi@yahoo.co.uk

For peer review only

ABSTRACT

Introduction: Depression is associated with negative patient outcomes for chronic diseases and likely affects consistent physical non-communicable diseases (NCDs) care management in relation to clinic attendance and medication adherence. We found no published studies on the integration of depression management in physical NCD clinics in Malawi and assessing its effects on patient and service outcomes. Therefore, the aim of this study is to evaluate the effectiveness of integrating depression screening and management in physical NCD routine care on patient and service outcomes in Malawi. We will also determine the sensitivity and specificity of the PHQ-9 in the detection of depression in NCDs clinic.

Methods and Analysis: The study will have two phases. Phase one will involve the validation of the PHQ-9 screening tool for depression, using a cross sectional study design involving 323 participants, in two specialised physical NCD clinics in one of the 28 districts of Malawi. Using a quasi-experimental study design in four districts of Malawi not involved in the Phase one study, Phase two study will evaluate the effectiveness of integrating depression screening (using PHQ-9) and management (based on a specially designed toolkit). Outcomes will be measured at three and six months among patients with comorbid diabetes (poorly controlled) and depression attending physical NCD clinics in Malawi.

Ethics and Dissemination: Ethical approval was obtained from the University of Malawi, College of Medicine Research and Ethics Committee (COMREC). The findings will be disseminated through presentations at journal clubs, senior management of the Ministry of Health, national and international conferences as well as submission to peer reviewed publications. Policy briefs will also be created.

For peer review only

Strengths and limitations of this study

- The cross sectional study design in phase one will allow us to estimate the prevalence of depression among people with diabetes**.**
- The quasi experimental design will enable us to investigate the impact of depression in determining the outcome of diabetes.
- The quasi-experimental design which will be used in second phase has a limitation that baseline differences between the clinics may lead to spurious results.

For peer review only

INTRODUCTION

Physical non communicable diseases and depression individually are common and significant causes of morbidity, disability and mortality in Sub Saharan Africa [1]. Non communicable diseases (NCDs) account for a growing burden on the population and health systems in low-and middle-income countries (LMIC), [2]. Similarly depression is projected to become a leading cause of disability by 2030 [3]. The World Health Survey of 2007 suggests that the prevalence of comorbidity of physical NCDs and depression is increasing; thus between 9.3% and 23.0% of people with NCDs had comorbid depression worldwide [4]. The global prevalence of major depression in people with diabetes is 27% [5]. The relationship between depression and physical NCDs is bidirectional. Modifiable risk factors for physical NCDs are exacerbated by poor mental health and on the other hand physical NCDs are a risk factor for mental illness[6]. Comorbidity is associated with a range of outcomes, including poor adherence or non-adherence to medication, functional impairment, increased medical costs, poor self-care regimens, increased medical symptom burden, increased morbidity and increased mortality [7 8 9]. The recent international study involving 14 countries shows that people with diabetes have depressive disorder as well as significant levels of depressive symptoms. Management of depression (pharmacological treatment combined with psychotherapy) in diabetes mellitus, has shown to be effective both on depression outcomes as well as diabetes management and glycaemic control[10 11].

Physical NCDs are common and in Malawi, it is estimated that NCDs account for at least 12% of total Disability Adjusted Life Years (DALYs) [12]. In addition, NCDs are the second leading cause of deaths in adults after AIDS [12]. Furthermore, a 2009 nationwide STEPwise approach to Surveillance (STEPS) survey showed that about 6% of the general population had diabetes mellitus [13].

Depression commonly overlaps with physical NCDs and depression complicates NCD care. We are not aware of any studies done in Malawi that assessed the burden of depression in physical NCDs care and its effect on diabetes outcomes. A recent study done in one of the 28 districts of Malawi, reported that control of diabetes is poor and there was high non adherence of NCDs clients to appointments [14]. Currently there are no published studies done in Malawi to explore factors associated with poor control of diabetes and the high rate of non-adherence to attending appointments in physical NCDs clinics.

In Malawi, there are few studies looking at depression screening and treatment in general care. The prevalence of depression among outpatient attendees in primary care clinics in Malawi in

For peer review only - http://bmjopen.bmj.com/site/about/guidelines.xhtml

2010 and 2013 ranged between 19 to 30% [15 16]. Despite the high prevalence of depression in general health care services in Malawi, the extent of comorbid depression in the context of NCDs is unclear. Using the results from general primary care clinics studies done in Malawi on detection rates for depression, it is likely that detection rates of depression in physical NCDs clinics in Malawi are poor. Undiagnosed and untreated depression more likely contributes to poor control of diabetes as shown in studies done in other countries [17-20]. Evidence from recent international study shows that identification and treatment of mental health problems is not routine in clinical care[20]. In Malawi, routine screening will assist in early detection of patients with co-morbid depression in physical NCD clinics and facilitate treatment. To our knowledge, there are no studies done in Malawi focusing on identification and interventions for depression in physical NCDs clinics. The integration of mental healthcare into other health services is one of the strategies in the National Mental Health Policy in Malawi[21]. The results of the proposed study will help to inform on decisions whether to integrate and the strategies for integrating depression management in physical NCD clinics in Malawi.

AIMS AND OBJECTIVES

Our overall objective is to evaluate the effectiveness of integrating screening and depression management into physical NCD routine care on patient and service outcomes in Malawi. In this way, we will contribute to evidence on comorbidity of depression and diabetes including the effects of identifying and treating depression in patients with comorbid diabetes and depression in physical NCD care in Malawi.

Our study has four specific objectives:

1. To determine the sensitivity and specificity of the PHQ-9 in the detection of depression in NCDs clinic,
2. To estimate the prevalence of comorbidity between depression and diabetes mellitus in Malawi,
3. To determine detection rate of depression in physical NCDs clinics in Malawi,
4. To compare patient outcomes in physical NCDs clinics with and without integration of depression screening and management.

RESEARCH QUESTIONS

The main research questions in this study are:

1. Will the validated Chichewa Patient Health Questionnaire (PHQ-9) demonstrate satisfactory sensitivity and specificity in physical NCD clinic settings in Malawi?
2. Will integrating depression screening using a validated PHQ-9 and depression

For peer review only

management (based on a specially designed toolkit) be more effective than usual care in identifying patients with comorbid depression and diabetes, facilitating treatment of depression in patients with comorbid depression and diabetes, and improving clinical outcomes among patients with comorbid depression and diabetes?

METHODS AND ANALYSIS

The study will be conducted from December 2017 to December 2018. The study will be in two phases. Phase One will be for validation of the PHQ 9 among diabetes patients. Phase two will be evaluating the effectiveness of integrating screening and depression management in patients with comorbid depression and diabetes attending physical NCD clinics. To answer the research questions, we will use a cross-sectional study design in Phase One and a quasi-experimental study design in Phase Two.

Patient and Public Involvement

Patients and or public were not involved in the design of this study however the results will be disseminated to study participants through the clinics Diabetes support groups.

Phase One: Validation of PHQ 9 in physical NCDs Clinic Setting in Malawi

Study design, area and physical NCD clinics

Depression varies based on cultural context as such screening tools must be adapted and validated for particular populations. PHQ-9 has not been validated in Malawi hence the study will assess validity of the PHQ-9. For Phase One, we will use a cross sectional study design. The study will be conducted in Lilongwe district which is one of the 28 districts in Malawi. Malawi is divided administratively into three regions namely Northern, Central and Southern. Lilongwe is located in the central region of Malawi. It has a population of 2,688, 379 people[22] with 8 physical NCD clinics.

The study will be conducted in two physical NCDs clinics (Area 25 Health Centre under Lilongwe District Health Office and Kamuzu Central Hospital). The Area 25 Health Centre serves a catchment population of 80, 000 while Kamuzu Central Hospital (a tertiary health facility) catchment population is the entire central region which is 7, 401,068 [22]. The physical NCD clinic at Area 25 Health Centre is staffed by clinical officers and nurses who have undergone appropriate training in the diagnosis and management of key NCDs while the clinic of Kamuzu Central Hospital is also staffed by medical officers (general medical doctors) and specialists on top of clinical officers and nurses. The two sites are implementing the World

For peer review only

Health Organization Package of Essential Non communicable diseases (WHO PEN) interventions. Figure 1 is a map of Malawi showing central region districts

(Insert figure 1 here)

SAMPLE SIZE DETERMINATION

The sample size was calculated based on sensitivity of PHQ 9 using Buderer's formula [23]. We used the following parameters; anticipated sensitivity (*SN*) was 80%, standard normal deviation corresponding to the specified size of the critical region ($z^2_{1-\alpha/2}$) is 3.84, alpha (*α)* which is size of the critical region is 0.05, L is the absolute precision desired on either side of sensitivity and was set at 0.1 and prevalence for depression was 20%. The prevalence of depression of 20% is based on a recent study on patients attending health care setting in Malawi [15]. We also considered potential participants' refusal and loss of data; consequently we put it at 5%. Taking these assumptions into consideration, the required sample size was calculated as 323.

RECRUITMENT AND DATA COLLECTION

Consecutive patients attending the NCD Clinic will be identified by research assistants. The research assistants will provide information about the study to the patients and seek informed written consent. Participants will be eligible for the study if they are at least 18 years of age and attending the NCDs clinic for diabetes care and available for an interview. Participants will be excluded if they require acute medical care or are unable to speak.

The research assistants will enrol patients who consent and carry out interviews at the health facility. When patients do not consent to participate in the study, the research assistant will request permission to record sex, age, level of education and occupation of the patient.
One research assistant will administer the PHQ 9 orally to ensure high and standard responses, due to low literacy of the study participants. The research assistant will then calculate the screening score and let the participant be attended to by the NCDs clinician. After the consultation the patient will have the Diagnostic and Statistical Manual of Mental Disorders (DSM–IV), Axis I disorders Clinical Version (SCID) administered by a second research assistant to elicit the psychiatric symptoms itemized in the Diagnostic and Statistical Manual of Mental Disorders (DSM) criteria for depression. The second research assistant will be masked from the PHQ-9 score. The training, supervision, and quality assurance of the research assistant doing the SCID will be done as well. A diagrammatic representation of the process is shown below in Figure 2.

(Insert figure 2 here)

For peer review only - http://bmjopen.bmj.com/site/about/guidelines.xhtml

MEASUREMENTS

The aim of Phase One is validating the Patient Health Questionnaire (PHQ-9) in physical NCDs clinics in order for it to be used for screening of depression in NCDs in Malawi. The PHQ-9 (based on DSM criteria) has been chosen for this study because it is brief, requires a short time to administer which is compatible with the Malawian health setting workload. Furthermore, the PHQ-9 can be used to establish depressive disorder diagnoses as well as depressive symptoms severity [24].

For screening data using PHQ-9, presence of depressive symptoms will be defined as having a total score of 5 and above on all the nine questions (PHQ-9 total score ≥ 5). Symptoms will be categorised using established cut-off points 5, 10 and 20 (mild, moderate, and severe depressive symptoms, respectively) [25 26]. The sensitivity and specificity at the standard cut-off point will be assessed, and performance at alternative cut-off point will also be examined to determine whether the standard cut-off point is optimal in this population

The SCID for depression will be used as the Gold standard to validate PHQ 9. The SCID for depression has been translated into Chichewa and has been used in Malawi after undergoing a process of validation which included translation, back translation and testing [27].

Data management

Data will be checked for consistency and completeness by the principal investigator and double entered to SPSS V.20 by experienced data entry clerks. Then, it will be cleaned and analysed using the same SPSS V.20. Hard copies of the data will be stored in a locked cabinet and consent forms will be separated from the data.

Data analysis

Data will be analysed using SPSS V.20. Descriptive statistics will be computed to describe the sociodemographic characteristics of participants and to summarise the frequencies of depression under each category in both criteria (PHQ-9 and SCID). We will assess whether refusal is associated with sociodemographic characteristics by testing the statistical significance of socio-demographic characteristics between refusals and consenters. The prevalence of diagnosed depression among patients with diabetes will be determined by computing the proportion of those with depression according to the SCID who will be correctly screened out by the PHQ.

The sensitivity, specificity and predictive values (PPV and NPV) of a PHQ-9 total score >=5,

For peer review only - http://bmjopen.bmj.com/site/about/guidelines.xhtml

with 95% confidence intervals, will be calculated relative to the reference standard of a diagnosis from the depression module of the SCID. A Receiver Operating Characteristic curve (ROC curve) will be made using the SPSS version 20 in order to get criterion validity of the PHQ-9. Internal consistency will be determined by Cronbach's alpha. In exploratory analyses we will examine the test characteristics of alternative thresholds.

Phase Two: Evaluation of the effectiveness of integrating screening using validated PHQ 9 and Depression management in physical NCD Clinics

Study design and setting

In Phase Two of the study, we will use a quasi experimental design employing a non-equivalent control group (pre-post) design. Prospective controlled interventional trials provide a higher level of evidence for a true causality [28]. The best choice of the controlled interventional studies is the true experimental design of which randomized controlled trial (RCT) is the gold standard. However nonrandomized control group trial is a reasonable choice when an RCT would be ideal but not due to costs and ethics limitations and also when trying to establish the effectiveness of large-scale dissemination and implementation [28 29]. Furthermore literature indicates the intervention often cannot be randomized for the following reasons: ethical considerations, an inability to randomize patients, an inability to randomize locations or settings, and a need to intervene quickly[29]. We have therefore chosen to use a quasi experimental design and the intervention program will be done in two district NCD clinics while the other two districts will provide usual care. Four convenient physical NCD clinics which are implementing the World Health Organization Package of Essential Non-communicable diseases (WHO PEN) interventions will be allocated to intervention arm (2 physical NCD clinics) and control arm (2 physical NCD clinics) and in each clinic, consecutive patients who meet the study criteria will be recruited.

The Phase Two of the study will be conducted for a period of 9 to 12 months in four health care facilities in Mchinji district, Kasungu district, Salima district and Dedza district of the Central region of Malawi (Figure 1). The four districts are also implementing the World Health Organization Package of Essential Non communicable diseases (WHO PEN) interventions. Currently each district has one physical NCD Clinic based at the District Hospital. The physical NCD clinic in the four districts are staffed by clinical officers and nurses who have undergone appropriate training in the diagnosis and management of key physical NCDs. They follow similar standard guidelines in the management of the physical NCDs.

Intervention

For peer review only

1. Clinicians at the physical NCD clinics will be trained in routine screening for depression with a validated PHQ 9,
2. Clinicians at the physical NCD clinics will be trained on comorbidity of diabetes and depression and coming up with individual care plans and prescribing and monitoring antidepressants
3. Clinicians at the physical NCD clinics will be trained on problem solving skills using the effective helping techniques for depression and how to make follow up on individual care plans.

Usual Care

This would involve the NCD clinicians treating patients with comorbid depression and diabetes using normal practices of treating depression involving antidepressants.

SAMPLE SIZE DETERMINATION

The sample size was based on detecting a difference in glycated haemoglobin at 6 months between study arms. The sample size was based on an Analysis of Covariance (ANCOVA) approach, with the outcome measurement at follow-up, and the baseline glycated haemoglobin considered as a covariate in the analysis. The correlation between baseline and follow-up haemoglobin values is estimated to be 0.5. A difference in glycated haemoglobin between arms of 0.7% is considered to be a clinically important difference (based on a previous study which found a difference of 0.56% [30]), and data from previous research suggested that the glycated haemoglobin measurements had a standard deviation of 1.8% [30]. It is calculated that using a 5% significance level and 80% power, 78 subjects per group are required. It is estimated that there will be a 10% drop out/loss to follow-up. To allow for this, we planned to recruit 87 per group, giving a total sample size for the study of 174 participants.

RECRUITMENT AND DATA COLLECTION

We will use a mixed methods (quantitative and qualitative) approach in Phase two. The reason for collecting both quantitative and qualitative data is to complement and also to address shortfall s of either approach. The use of both forms of data allows researchers to gain deeper understanding of the phenomenon under study [31]. In addition, evidence suggests that triangulation of methods increases both the validity and reliability of data [32].

The research assistant with the help of health professionals running the NCDs clinic at each site will identify the study participants who meet the inclusion criteria consecutively. The research assistants will provide information about the study to the patient and seek written informed

For peer review only - http://bmjopen.bmj.com/site/about/guidelines.xhtml

consent. Participants will be eligible for the study if they are at least 18 years of age and attending the NCDs clinic for diabetes care and available for an interview. Participants will be excluded if they require acute medical care or are unable to speak. The research assistants will enrol patients who consent and carry out interviews at the health facility. When patients do not consent to participate in the study, the research assistant will request permission to record sex, age, level of education and occupation of the patient. Adult patients with poorly controlled diabetes (a glycated hemoglobin level of 8.5% or higher as suggested from previous research [30 33]) and comorbid depression will be recruited for the study and followed up to 6 months after recruitment (Figure 3). Data collection will occur at three time-points: baseline, at 3 months and at 6 months.

Qualitative interviews will be conducted among health care workers in order to assess feasibility and acceptability of integrating depression management into NCDs care. In-depth interviews will be employed to understand health professionals' experience in both using the validated PHQ-9 for screening and the depression management guidelines.

(Insert figure 3 here)

MEASUREMENTS

The primary exposure variable

Depression will be measured using the PHQ-9. The PHQ-9 will have been validated in Phase One of this study.

The exposure of interest is whether the patient is receiving care at an intervention or usual care facility.

Outcome variables

Primary outcome variable

The patient diabetes outcome for this study is blood glucose control. Data on the patients' status on the primary outcome will be extracted from the NCD patient mastercard. For the purpose of the study in checking how well diabetes is being controlled, glycated haemoglobin (HbA1c) test will be performed on the recruited study participants at baseline, 3 and 6 months. Depression is the co-primary outcome. The PHQ-9 will be administered at baseline, 3 and 6 months.

Secondary outcome variable

For peer review only

Quality of life (QoL) will be measured using the interviewer administered version of the EuroQol 5 dimensions questionnaire (EQ-5D). This instrument has been validated in South Africa and Zimbabwe [34 35]. The tool has been translated into several languages including Afrikaners, Xhosa and Shona [35]. The EQ-5D will be adapted in Malawi and will be used at baseline and on follow up of patients at three and six months.

Data management

Data will be checked for consistency and completeness by the principal investigator and double entered to SPSS V.20 by experienced data entry clerks. Hard copies of the data will be stored in a locked cabinet and consent forms will be separated from the data.

Data analysis

The primary outcome is glycated haemoglobin at 6 months. This will be compared between groups using Analysis of Covariance (ANCOVA). The haemoglobin value at baseline will be considered as a covariate in the analysis.

The glycated haemoglobin values at 3 months will be considered as a separate outcome, and analysed using equivalent methods to the primary outcome.

Quantitative data will be analyzed statistically using a statistical package for social sciences version 20 (SPSS-20). Descriptive analysis will be done. The recorded qualitative data on the feasibility and acceptability of integrating depression management will be transcribed and coded using Atlas ti. Relevant queries will be created in the database to thematically analyse the data.

Ethical considerations

The protocol was approved by the University of Malawi, College of Medicine Research and Ethics Committee (COMREC) on 31st August 2017 (Reference-P.07/17/2218). Written informed consent will be obtained from every participant and fingerprint impressions will be taken from consenting illiterate participants. Patients identified as having depression or endorsing the suicide item on PHQ-9, will be referred to appropriate professionals for further assessment and management. A permission to conduct the study has been sought from the District Health Officers of Lilongwe, Kasungu, Dedza, Mchinji, Salima and Hospital Director of Kamuzu Central Hospital. Study participants will not be given any financial reimbursement, however they will thanked verbally for their participation. Privacy will be maintained as the interviews will be conducted in a room which will provide both visual and audio privacy. Referrals will be made to appropriate professionals for further assessment and management in cases where participants will be identified to have depression as well as those that will screen

For peer review only

positive for suicidal ideations through the interviews.

Dissemination plan

The findings will be disseminated through publications in peer-reviewed journals and conference presentations. Summary reports will be submitted to the participating institutions and to the University of Malawi, College of Medicine. The results will also be shared with policy makers and other local stakeholders in form of policy briefs as part of evidence on integration of depression management in NCDs.

Limitations of the study

In this study, people may have undiagnosed comorbid illnesses and our method of screening may not be strong in capturing other comorbid illnesses. In the first phase of validation we will use two specialized clinics which may not be representative of the wider population. Quality of life will be measured using five domains and we will therefore not obtain detailed information on the various dimensions that assess health-related quality of life (HRQoL). The quasi-experimental design which will be used in second phase has also a limitation that baseline differences between the clinics may lead to spurious results.

Strengths of the study

The study will provide much needed evidence about the impact of comorbid depression on the outcome of diabetes. The cross sectional study design will allow us to estimate the prevalence of depression among people with diabetes. The study will enable us to investigate the impact of depression in determining the outcome of diabetes. To our knowledge, this study will be the first in Malawi, to examine identification and interventions for depression in physical NCD clinics.

Expected benefits of the findings

Our findings will contribute to sparse evidence on mental health, diabetes and other NCDs in low- income and middle-income countries. We anticipate that our study findings will raise awareness of comorbidity among clinicians, service providers, policy makers and that it will contribute to clinical practice and policy change regarding the delivery of mental healthcare in NCDs care in Malawi.

Authors' contributions

For peer review only

MU, BP, FK and AM were involved in the conceptualization of the study. MU was responsible for drafting the protocol manuscript. BP, FK and AM edited the protocol. All authors read and approved the final manuscript.

Funding statement

This study protocol presents independent work supported through the DELTAS Africa Initiative [DEL-15-01]. The DELTAS Africa Initiative is an independent funding scheme of the African Academy of Sciences (AAS)'s Alliance for Accelerating Excellence in Science in Africa (AESA) and supported by the New Partnership for Africa's Development Planning and Coordinating Agency (NEPAD Agency) with funding from the Wellcome Trust [DEL-15-01] and the UK government. The views expressed in this publication are those of the author(s) and not necessarily those of AAS, NEPAD Agency, Wellcome Trust or the UK government.

Competing interests statement

The authors declare that they have no competing interests.

For peer review only

Reference

1. Nikolic IA, Stanciole AE, Zaydman M. Chronic emergency: why NCDs matter. Health, Nutrition, and Population Discussion Paper 2011.
2. Buttorff C, Hock RS, Weiss HA, et al. Economic evaluation of a task-shifting intervention for common mental disorders in India. Bulletin of the World Health Organization 2012;**90**(11):813-21.
3. Mathers C, Fat DM, Boerma JT. The global burden of disease: 2004 update. Geneva, Switzerland: World Health Organization. 2008.
4. Moussavi S, Chatterji S, Verdes E, et al. Depression, chronic diseases, and decrements in health: results from the World Health Surveys. The Lancet 2007;**370**(9590):851-58.
5. World Health Organization. Investing in mental health. Geneva: World Health Organization, 2003.
6. Ngo VK, Rubinstein A, Ganju V, et al. Grand challenges: integrating mental health care into the non-communicable disease agenda. PLoS Med 2013;**10**(5):e1001443.
7. Mensah GA, Collins PY. Understanding mental health for the prevention and control of cardiovascular diseases. Global heart 2015;**10**(3):221-4.
8. Rieckmann N, Gerin W, Kronish IM, et al. Course of depressive symptoms and medication adherence after acute coronary syndromes: an electronic medication monitoring study. J Am Coll Cardiol 2006;**48**(11):2218-22.
9. Gehi A, Haas D, Pipkin S, et al. Depression and medication adherence in outpatients with coronary heart disease: findings from the Heart and Soul Study. Arch Intern Med 2005;**165**(21):2508-13.
10. van der Feltz-Cornelis CM, Nuyen J, Stoop C, et al. Effect of interventions for major depressive disorder and significant depressive symptoms in patients with diabetes mellitus: a systematic review and meta-analysis. Gen Hosp Psychiatry 2010;**32**(4):380-95.
11. Simon GE, Katon WJ, Lin EH, et al. Cost-effectiveness of systematic depression treatment among people with diabetes mellitus. Arch Gen Psychiatry 2007;**64**(1):65-72.
12. MoH, WHO. Malawi National STEPS Survey for Chronic Non-Communicable Diseases and their Risk Factors, 2010.
13. Msyamboza KP, Ngwira B, Dzowela T, et al. The burden of selected chronic non-communicable diseases and their risk factors in Malawi: nationwide STEPS survey. PLoS One 2011;**6**(5):e20316.
14. Manjomo RC, Mwagomba B, Ade S, et al. Managing and monitoring chronic non-communicable diseases in a primary health care clinic, Lilongwe, Malawi. Public Health Action 2016;**6**(2):60-65.
15. Kauye F, Jenkins R, Rahman A. Training primary health care workers in mental health and its impact on diagnoses of common mental disorders in primary care of a developing country, Malawi: a cluster-randomized controlled trial. Psychological medicine 2014;**44**(03):657-66.
16. Udedi M. The prevalence of Depression among patients and its detection by primary health care workers at Matawale Health Centre (Zomba). Malawi Medical Journal 2014;**26**(2):34-37.
17. Ciechanowski P, Russo J, Katon W, et al. Where is the patient? The association of psychosocial factors and missed primary care appointments in patients with diabetes. General hospital psychiatry 2006;**28**(1):9-17.
18. Andreoulakis E, Hyphantis T, Kandylis D, et al. Depression in diabetes mellitus: a comprehensive review. Hippokratia 2012;**16**(3):205-14.
19. Lin EHB, Rutter CM, Katon W, et al. Depression and Advanced Complications of Diabetes. Diabetes Care 2010;**33**(2):264.
20. Lloyd CE, Nouwen A, Sartorius N, et al. Prevalence and correlates of depressive disorders in people with Type 2 diabetes: results from the International Prevalence and Treatment of Diabetes and Depression (INTERPRET-DD) study, a collaborative study carried out in 14 countries. Diabet Med 2018.

For peer review only

21. Ministry of Health and Population. National Mental Health Policy. Lilongwe, Malawi: Ministry of Health and Population, 2001.
22. National Statistical Office (NSO). Population Projections Malawi. Zomba, Malawi: National Statistical Office (NSO), 2009.
23. Buderer NMF. Statistical methodology: I. Incorporating the prevalence of disease into the sample size calculation for sensitivity and specificity. Academic Emergency Medicine 1996;**3**(9):895-900.
24. Kroenke K, Spitzer RL, Williams JBW. The PHQ-9: Validity of a Brief Depression Severity Measure. Journal of General Internal Medicine 2001;**16**(9):606-13.
25. Lowe B, Kroenke K, Herzog W, et al. Measuring depression outcome with a brief self-report instrument: sensitivity to change of the Patient Health Questionnaire (PHQ-9). Journal of affective disorders 2004;**81**(1):61-66.
26. Lowe B, Unutzer J, Callahan CM, et al. Monitoring depression treatment outcomes with the patient health questionnaire-9. Medical care 2004;**42**(12):1194-201.
27. Stewart RC, Kauye F, Umar E, et al. Validation of a Chichewa version of the self-reporting questionnaire (SRQ) as a brief screening measure for maternal depressive disorder in Malawi, Africa. Journal of Affective Disorders 2009;**112**(1-3):126-34.
28. Axelrod DA, Hayward R. Nonrandomized Interventional Study Designs (Quasi-Experimental Designs). In: Penson DF, Wei JT, eds. Clinical Research Methods for Surgeons. Totowa, NJ: Humana Press, 2007:63-76.
29. Eliopoulos GM, Harris AD, Bradham DD, et al. The use and interpretation of quasi-experimental studies in infectious diseases. Clinical Infectious Diseases 2004;**38**(11):1586-91.
30. Katon WJ, Lin EH, Von Korff M, et al. Collaborative care for patients with depression and chronic illnesses. New England Journal of Medicine 2010;**363**(27):2611-20.
31. Creswell JW. *Research design: Qualitative, quantitative, and mixed methods approaches*: Sage publications, 2013.
32. Ajayi I, Olumide E, Oyediran O. Patient satisfaction with the services provided at a general outpatients' clinic, Ibadan, Oyo State, Nigeria. African journal of medicine and medical sciences 2005;**34**(2):133-40.
33. Wilkinson JE, Culpepper L. Peer Reviewed: Associations Between Colorectal Cancer Screening and Glycemic Control in People With Diabetes, Boston, Massachusetts, 2005-2010. Preventing chronic disease 2011;**8**(4).
34. Jelsma J, Mhundwa K, De Weerdt W, et al. The reliability of the Shona version of the EQ-5D. The Central African journal of medicine 2001;**47**(1):8-13.
35. Jelsma J, Ferguson G. The determinants of self-reported health-related quality of life in a culturally and socially diverse South African community. Bulletin of the World Health organization 2004;**82**(3):206-12.

For peer review only

Figures

Figure 1: Map of Malawi showing central region districts and study sites

Figure 2: Phase One study procedure

Figure 3: Phase Two study procedure

For peer review only

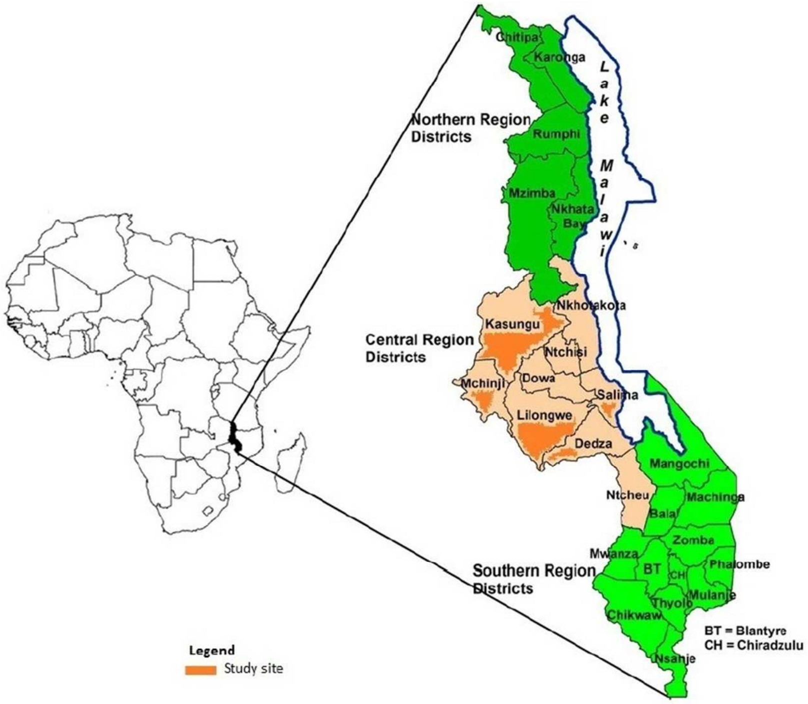

Figure 1: Map of Malawi showing central region districts and study sites

85x65mm (300 x 300 DPI)

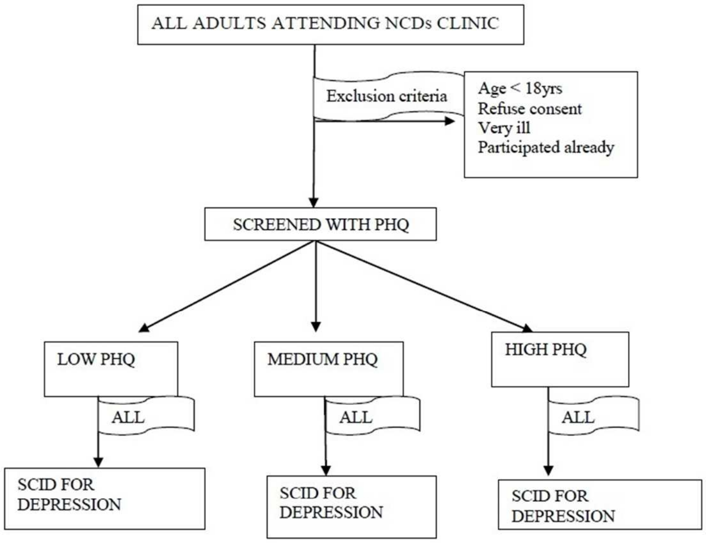

Figure 2: Phase One study procedure

87x56mm (300 x 300 DPI)

view only

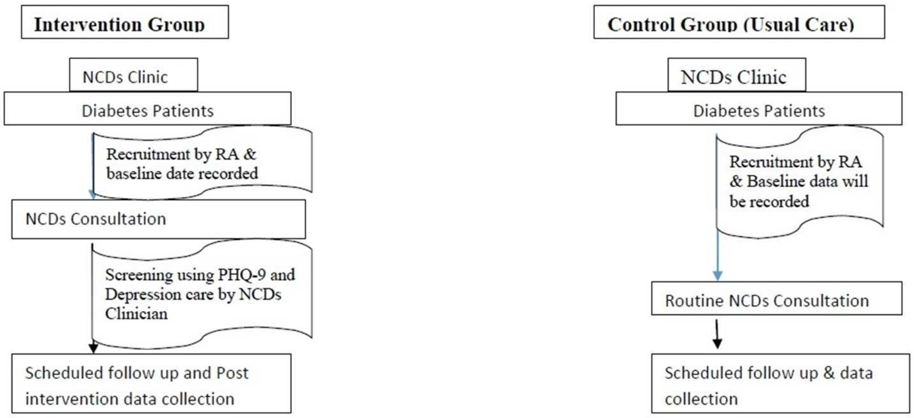

**Three months follow up**		**Three months follow up**
PHQ 9 score		PHQ 9 score
Diabetes control check		Diabetes control check
EQ-5D		EQ-5D
**Six month follow up**		**Six month follow up**
PHQ 9		PHQ 9
Diabetes control check		Diabetes control check
EQ-5D		EQ-5D
**Baseline assessments will be done for all three measures & recorded**		**Baseline assessments will be done for three measures only & recorded**

Figure 3: Phase Two study procedure

88x87mm (300 x 300 DPI)
